# Familiarity to a Feed Additive Modulates Its Effects on Brain Responses in Reward and Memory Regions in the Pig Model

**DOI:** 10.1371/journal.pone.0162660

**Published:** 2016-09-09

**Authors:** David Val-Laillet, Paul Meurice, Caroline Clouard

**Affiliations:** INRA, UR1341 ADNC, St Gilles, France; The University of Tokyo, JAPAN

## Abstract

Brain responses to feed flavors with or without a feed additive (FA) were investigated in piglets familiarized or not with this FA. Sixteen piglets were allocated to 2 dietary treatments from weaning until d 37: the naive group (NAI) received a standard control feed and the familiarized group (FAM) received the same feed added with a FA mainly made of orange extracts. Animals were subjected to a feed transition at d 16 post-weaning, and to 2-choice feeding tests at d 16 and d 23. Production traits of the piglets were assessed up to d 28 post-weaning. From d 26 onwards, animals underwent 2 brain imaging sessions (positron emission tomography of ^18^FDG) under anesthesia to investigate the brain activity triggered by the exposure to the flavors of the feed with (FA) or without (C) the FA. Images were analyzed with SPM8 and a region of interest (ROI)-based small volume correction (p < 0.05, *k* ≥ 25 voxels per cluster). The brain ROI were selected upon their role in sensory evaluation, cognition and reward, and included the prefrontal cortex, insular cortex, fusiform gyrus, limbic system and corpus striatum. The FAM animals showed a moderate preference for the novel post-transition FA feed compared to the C feed on d 16, *i*.*e*., day of the feed transition (67% of total feed intake). The presence or absence of the FA in the diet from weaning had no impact on body weight, average daily gain, and feed efficiency of the animals over the whole experimental period (p ≥ 0.10). Familiar feed flavors activated the prefrontal cortex. The amygdala, insular cortex, and prepyriform area were only activated in familiarized animals exposed to the FA feed flavor. The perception of FA feed flavor in the familiarized animals activated the dorsal striatum differently than the perception of the C feed flavor in naive animals. Our data demonstrated that the perception of FA in familiarized individuals induced different brain responses in regions involved in reward anticipation and/or perception processes than the familiar control feed flavor in naive animals. Chronic exposure to the FA might be necessary for positive hedonic effects, but familiarity only cannot explain them.

## Introduction

In many species including humans, food intake can be stimulated by the use of food additives. These dietary substances can modulate the food organoleptic properties and consequently food palatability, as observed for specific flavors [1], but they can also influence satiety signals *via* the modification of post-ingestive visceral information [2]. Many authors have described the positive impact of feed additives such as flavors to improve feed intake and growth in piglets during post-weaning feed transitions [3–6]. The identification of food additives susceptible to modify the appetite and/or hedonic value of food could open the way to many applications in the domain of human and animal nutrition. In human nutrition, additives capable of stimulating food (or medicine) intake might allow the development of potent strategies to alleviate or treat some eating disorders and food aversions encountered in clinics, but also improve the palatability of pediatric medications for example. In animal nutrition, the characterization of eating behavior modifiers might help to support or even improve feed intake during critical feed transitions or stressful conditions, notably in reproductive sows and growing piglets.

Recent studies demonstrated that the pig model is particularly adapted to explore the behavioral and neurobiological consequences of conditioned flavor preferences and aversions, and more generally to study the brain responses to food stimuli [7, 8], especially in neural networks involved in the characterization of food palatability, food motivation, reward expectancy and food control in the human. For example, exposure to preferred flavors induced a higher activity in corticolimbic and reward-related areas, while aversive flavors induced a deactivation of the basal nuclei and limbic thalamic nuclei [8]. Clouard *et al*. [7] also showed a global deactivation of the prefrontal cortex, specific activation of the posterior cingulate cortex, as well as asymmetric brain responses in the basal nuclei and temporal gyrus in pigs exposed to aversive or less preferred flavors. These results indicate that exteroceptive stimuli (*e*.*g*. taste and odor) have a major role in recalling memories of the learned food sensory image and the associated individual experience, which mediate food-related cognitive processes (*i*.*e*. choice, decision to eat or not).

In a previous study performed in our lab, the absolute and relative preferences of piglets for 8 sensory feed additives have been investigated [9]. The additives with the highest effect on relative feed preference were selected in this preliminary work and further tested in the context of a feed transition. The addition of these compounds in an unfamiliar solid feed led to increased feed intake on the day of transition, which suggests a positive effect of these additives on appetite under stress conditions in the piglet [9]. In a more recent study [10], feed additives were added to a pre-transition diet from weaning onwards, and in an unfamiliar post-transition diet after feed transition to evaluate the impact of early familiarization with the additives on feed preferences during the early post-weaning period. One of these additives increased acceptance of the unfamiliar post-transition diet on the day of feed transition but also at least 10 days after the transition, highlighting a long-term effect on feed preference.

These data raise the question of the neurobiological mechanisms underlying the modulating effects of these additives on appetite and food pleasure. Are these additives able to modulate the brain reward circuit to influence food choices and motivation? Are these effects only dependent on habituation and familiarization processes, therefore acting *via* memory processes that recall sensory information related to food ingestion and the pleasure to eat? Or rather, is there an intrinsic impact of these sensory additives on the brain reward circuit and if so, to which extent this property could be applied in human nutrition and/or animal production? The aim of this study was thus to investigate the brain responses to feed flavors with or without a functional feed additive in piglets that have been familiarized to this additive from weaning onwards or not. The brain regions of interest were selected upon their role in learning, memory, food reward evaluation, emotions and cognitive control [11, 12], and included the prefrontal cortex and insular cortex, the fusiform gyrus, the limbic system (hippocampus, cingulate, entorhinal, perirhinal and parahippocampal cortices, amygdala and prepyriform area) and the corpus striatum (putamen, caudate, nucleus accumbens and globus pallidus).

## Materials and Methods

The experiment presented in this paper was conducted in accordance with the current ethical standards of the European Community (Directive 2010/63/EU), Agreement No. A35-622 and Authorization No. 35–88. The Regional Ethics Committee in Animal Experiment of Brittany has validated the entire procedure described in this paper and specifically approved this study (R-2012-DVL-02).

### Animals and housing

Four batches of 4 Large White/Landrace × Piétrain female piglets from the experimental station of the French National Institute of Agricultural Research (INRA, Saint Gilles, France) were used. Piglets, which weighed 8.81 ± 0.14 kg at the beginning of the study, were weaned at 28.31 ± 0.23 days of age and housed in individual pens (80 × 60 × 68 cm) equipped with a 2-part feeding trough for the implementation of 2-choice feeding tests. The room temperature was kept at 23.39 ± 0.08°C with a 13:11-h light-dark cycle.

### Experimental meals and feed beverages

One sensory feed additive (FA) was tested in the present study. This product was provided by a commercial company specialized in sensory functional food formulation (Phodé Laboratories, Terssac, France) and was mainly made of a natural extract of *Citrus sinensis* (60–80%).

The pigs were fed a first stage pelleted pre-transition diet from weaning (d 1) to 15 days after weaning (d 15) and a second stage pelleted post-transition diet from d 16 to the end of the study. The diets were formulated so that the nutrient composition (other than TSAA) met or exceeded recommendations of the NRC (1998) throughout the experimental period (**Table 1**). Two experimental diets were formulated by the adjunction of the FA in the pre- and post-transition pelleted diets. The powdered FA was incorporated in a matrix of wheat middling and the resulting FA/wheat middling blend was added in the pre- and post-transition diets during the feed formulation process at 10 kg/t of feed, so as the inclusion rate of the FA was 0.003125‰ (w/w). The inclusion rate was chosen by our industrial partner (Phodé Laboratories) and was supported by unpublished data as well as previous findings obtained in our laboratory [9, 10]. Additionally, 2 control (C) diets were formulated by the adjunction of the matrix of wheat middling with no additive at 10 kg/t of feed in the pre- and post-transition diets.

**Table 1:** Composition of the pre- and post-transition diets (as-fed basis)^1^.

		Pre-transition diet	Post-transition diet
Ingredient composition, %			
	Wheat	–	23.20
	Corn	–	25.00
	Barley	45.31	24.05
	Soybean meal	17.50	22.57
	Soybean proteins	2.50	–
	Vegetal oil	2.30	0.45
	Mild lactoserum	20.00	–
	Fattened milk	8.00	–
	Carbonate calcium	1.41	1.13
	Monocalcic phosphate	0.80	0.97
	Salt	–	0.40
	Vitamin and mineral premix^2^	0.50	0.50
	L-Lysine HCl	0.41	0.78
	DL-Methionine	0.26	0.20
	L-Tryptophan	0.46	0.43
	L-Threonine	0.17	0.17
	L-Valine	0.07	0.04
	Acidifying agent	0.10	0.10
	Phytase	0.20	–
Analyzed composition and nutritional value^3^			
	CP, %	18.99	18.00
	NDF, %	10.62	13.11
	Minerals, %	7.02	5.44
	NE, MJ/kg	10.63	9.67
	ME, MJ/kg	13.92	12.99

^1^ Piglets were fed the pre-transition diet from d 1 (weaning) to 15 and the post-transition diet from d 16 to the end of the experiment.

^2^ Supplied per kilogram of diet (as-fed basis): vitamin A, 10,000 IU; vitamin D3, 2,000 IU; vitamin E, 20 mg; vitamin K3, 2 mg; thiamine, 2 mg; riboflavin, 5 mg; niacin, 20 mg; pantothenic acid, 10 mg; pyridoxine, 5 mg; biotin, 0.2 mg; folic acid, 1 mg; vitamin B12, 0.03 mg; choline chloride, 600 mg; ascorbic acid, 40 mg; Fe, 100 mg; Cu, 20 mg; Zn, 100 mg; Mn, 40 mg; I, 0.6 mg; Se, 0.3 mg; and Co, 1 mg.

^3^ CP, crude protein; NDF, neutral detergent fiber; NE, net energy; ME, metabolisable energy.

Two feed solutions which had the flavor of the 2 experimental post-transition diets were formulated to perform the olfactogustatory stimulation during brain imaging and investigate the brain metabolism triggered by the exposure to the flavors of the C and FA post-transition diets. The C pelleted post-transition diet was crushed into a powder using a grinder (GRINDOMIX Retsch GM200; 20 s, 10 000 rpm). The resulting C feed powder was diluted at 10% (w/v) in tap water. After 12 h of settling, the supernatant was collected and resulted in the C feed solution. The dilution was chosen according to previous unpublished findings obtained in our laboratory and showing that at this dilution, the flavor of the feed was discriminated by both humans and pigs. In pigs, this flavor was significantly more attractive than water (*n* = 8, p < 0.01). As the FA powder included in the FA post-transition diet was not water-soluble, the FA feed solution could not be formulated using the same procedure. Consequently, the FA feed solution was formulated by the adjunction of a liquid water-soluble form of the FA in the C feed solution. The liquid water-soluble FA was first diluted in distilled water at 1% (w/w). Then, the solution was diluted in the feed beverage at 10 g/L. The inclusion rate of the FA in the C feed solution was fixed at 0.1% and was chosen according to data obtained by Phodé Laboratories (unpublished data) so as to ensure that the FA flavor had the same intensity as in the FA post-transition diet.

### Experimental procedure

**Fig 1:**
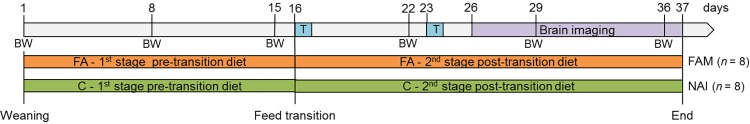
Experimental design. All animals were fed a first stage pre-transition diet from weaning to d 15, with (FA) or without (C) additive. On d 16, a feed transition was performed and the pre-transition diet was replaced by a post-transition diet of different composition, with or without the same additive. During the 2-choice tests (T) performed on d 16 and d 23, the naive (NAI) and familiarized (FAM) piglets had the choice between the post-transition diet without additive and the same diet with the additive. Body weight was assessed weekly (BW) and, from d 26 onwards; animals were subjected to 2 brain imaging sessions to assess the brain metabolism triggered by the exposure to the flavor of the C or FA post-transition diet.

The experimental timeline is described in **Fig 1**.

#### Feed intake and growth

On the weaning day (d 1), the animals were divided into 2 experimental groups of equivalent mean weight, with 2 animals per group in each batch (*n* = 8 animals per group in total; familiarized group: 8.86 ± 0.21 kg; naive group: 8.75 ± 0.21 kg). From weaning (d 1) to 15 days after weaning (d 15), the pigs were fed the pre-transition diet corresponding to their treatment (*i*.*e*. FA or C pre-transition diet) and from d 16 (day of feed transition) to the end of the study (d 37 on the latest), the pigs were fed the post-transition diet corresponding to their treatment (*i*.*e*. FA or C post-transition diet)–except on the 2-choice feeding tests days, that is on d 16 and d 23 where the animals were offered simultaneous access to the FA and C post-transition diets (*see below*). Each day, the animals had a 24-h access to feed, that is from 0900 h to 0900 h the next day. Throughout the experiment, the amount of feed distributed per day was adjusted so as to ensure that no feeder was emptied at the end of the working day, that is at 1700 h. The daily feed ration was distributed in small portions and several times during the day to prevent feed wastage and contamination by urine and defecations. The daily feed refusals were weighed every day at 0900 h, and the piglets were weighed weekly on days 1, 8, 15, 22, 29 and 36 before the daily feed provision. Average daily feed intake (ADFI, g/day), average daily gain (ADG, g/day), and gain to feed ratio (G:F, *i*.*e*. a measure of feed efficiency) were calculated for each experimental group.

#### Two-choice feeding tests

On d 16 (*i*.*e*. the day of the feed transition) and d 23 (*i*.*e*. 7 days after the feed transition), the animals were subjected to 2 consecutive 2-choice feeding tests: a 1-h 2-choice test from 0900 h to 1000 h and a 6-h 2-choice feeding test from 1000 h to 1600 h. These tests were carried out to investigate the pigs’ preferences during a short period and a longer period of exposure to the diets between the C post-transition diet and the FA post-transition diet. The exposure period was not prolonged over 7 h in order to minimize the contact of naive animals with the additive prior brain imaging.

On the days of the tests, the daily refusals of the day before were weighed at 0800 h before the pigs were subjected to the 1-h and 6-h 2-choice tests. For each test, the 2 post-transition diets were distributed in equal quantities in each compartment of the 2-part trough. The feeds were distributed once at 0900 h during the 1-h 2-choice tests, and then at 1000 h and 1400 h during the 6-h 2-choice tests. The refusals were weighed at the end of each test. Left and right positions of the diets in the troughs were counterbalanced across animals and test days to prevent any laterality bias.

#### Brain imaging procedure

From d 26 after weaning, the animals underwent 2 brain-imaging sessions to investigate the brain metabolism triggered by the exposure to the flavor of the C and FA post-transition diets. The brain imaging modality used to investigate the regional cerebral glucose metabolism (rCGM) was the PET of ^18^F- fluorodesoxyglucose (^18^F-FDG, CIS bio international, France).

Animal preparation and olfactogustatory stimulation. After fasting overnight for 15–18 h, the animals were sedated using Ketamine (15–20 mg/kg, Mérial, Lyon, France) given intramuscularly in the neck. Suppression of pharyngotracheal reflex was obtained by inhalation of isoflurane (5% v/v, Baxter SAS, Maurepas, France) immediately before tracheal intubation. The level of anesthesia was maintained by isoflurane (2% v/v) so as the Minimum Alveolar Concentration (MAC) remained at 1.8 during the whole procedure. Heart and respiratory rates were continuously monitored during anesthesia using a pulse oxymeter (Ohmeda oxymeter, GE Healthcare Clinical Systems, Limonest, France) and ECG electrodes (Immed Europe, Ennevelin, France). The animals were placed in a head first supine position on the bed of a whole body, high-resolution PET and a venous catheter was inserted into their left ear in order to inject the radiolabel. The ears and eyes of the animals were sealed with cotton and surgical tape respectively, in order to minimize auditory and visual stimulations. Animals’ body temperature was maintained at least at 37°C by using a heating blanket.

The olfactory stimulation consisted in diffusing an odorized air into the pig’s left snout (4 L/min). As the animals were intubated and mechanically ventilated, the diffused air could not come out from the mouth. Consequently, the olfactory stimulation was performed *via* one of the two snouts to let the air flow through the nasal cavity. The choice to perform the stimulation *via* the left snout rather than the right snout, however, has been done arbitrarily. A tube was inserted in the left snout of the animal and connected to a device designed in our laboratory (for an illustration of the apparatus, see [7]) and composed of a medical air cylinder connected to a flow meter and a two-way circuit of bottles equipped with a system of electronic valves. One of the bottles contained tap water (for neutral stimulation) and the other contained the FA or C feed solutions. Air was injected in the liquids contained in the bottles, which induced an extraction of the aromatic compounds of the beverages and generated an odorized air. The gustatory stimulation consisted in irrigating the pig’s tongue (24 mL/min) with tap water (for neutral stimulation) or the FA or the C feed solutions. A tube was positioned on the middle of the tongue and connected to a computer-operated automat developed in our laboratory (Gustautomat, INRA, St Gilles, France, see [7, 8]) and inspired by the Taste–o–Matic designed by Hellekant’s group [13]. The animals were subjected to a neutral olfacto-gustatory stimulation (i.e. diffusion of air in the snout and tap water on the tongue) for 5 min to accommodate the mucosa thermoreceptors and mechanoreceptors to the stimulation [14]. Then, stimulation with the FA or C feed flavors was performed for 15 min by the diffusion of odorized air in the snout and feed solutions on the tongue of the pig. The stimulation was ended by a 15-min neutral stimulation.

#### Data acquisition

The ^18^fluoro-deoxyglucose radiolabel (^18^F-FDG, 200 MBq) was injected through the venous catheter 5 min after the beginning of the olfactogustatory stimulation procedure. Brain images were acquired on a CTI/ Siemens HR+ tomograph in 3D mode (Siemens ECAT, 962, HR+). A 30-min emission scan was started 40 min after the radiolabel injection using an axial FOV of 15.52 cm. The effects of radiation self-attenuation were corrected by a 15-min transmission scan using an external positron-emitting isotope (^68^Ge). Following scatter, dead time and random corrections, the emission data were reconstructed using a ramp filter (6 mm full width half-maximum, 63 contiguous slices). Spatial resolution after reconstruction was 0.64 mm per pixel in the x and y directions and 2.42 mm per pixel in the z-axis. Pixel depth encoding was performed using the Standard Uptake Value (SUV) method.

#### Image processing

DICOM data were converted in NIfTI data with AMIDE-bin 1.0.2 (ed. Andreas Leoning) and were preprocessed and analyzed with statistical parametric mapping (SPM8, Wellcome Trust Centre for Neuroimaging, London, UK) implemented in MATLAB 7.1 (The Mathworks Inc., Natick MA, USA). SPM8 was adapted to the characteristics of the pig brain. The images were first manually reoriented and the spatial coordinates were centered compared to a reference point (x_0_, y_0_, z_0_, posterior commissure). The images were masked to remove the extracerebral matter, the coordinates were realigned to the mean and the images were spatially normalized. An affine transformation was performed to determine the 12 optimum parameters for registering the brain images a template and the subtle differences between the transformed image and the template were then removed using a non-linear registration method. Spatially normalized images were subsequently smoothed with a 4 × 4 × 4 mm full width at half maximum Gaussian kernel. Finally, a second narrower masking was performed to eliminate more finely the extracerebral matter. Eleven male and female pigs of approximately 35 kg different from those used in this experiment were used to build the PET template. PET images were acquired in the same injection, acquisition and reconstruction conditions.

### Statistical analysis

#### Statistical behavior analysis

Statistical analyses were performed with the R 2.14.1 software (The R Foundation for Statistical Computing, Vienna, Austria). Data were tested for normal distribution and homogeneity of variances. Performance data (ADG, ADFI, G:F, initial and final BW) were compared between groups with Student’s *t* tests. The feed preferences during the 2-choice tests were analyzed using paired and unpaired *t*-tests. All data are reported as means ± SEMs. Feed preferences were also expressed as the percentage of the FA feed intake relative to total intake. According to the scale of Goatcher and Church described in Kennedy and Baldwin [15], a percentage of feed intake from 60 to 80% of total feed intake indicates a moderate preference, and from 80 to 100%, a strong preference for the feed. The level of significance for all analyses was set as p < 0.05 and trends were considered at 0.05 < p ≤ 0.15.

#### Statistical brain image analysis

The regional ^18^F-FDG uptake was standardized to the mean global uptake using proportional scaling in order to minimize inter-individual differences in global CGM. Images were entered into a mixed between- and within-subjects 2 × 2 ANOVA [(familiarized group *vs*. naive group) by (stimulation with FA feed flavor *vs*. stimulation with C feed flavor)]. The following abbreviations are used in the manuscript: familiarized group (FAM), naive group (NAI), feed flavor with additive stimulation (SFA), control feed flavor stimulation (SC). *Post hoc* comparisons were conducted using independent or paired *t* tests by analyzing the following contrasts: [(NAI–SC) *vs*. (NAI–SFA)], [(FAM–SFA) *vs*. (FAM–SC)], [(FAM–SC) *vs*. (NAI–SFA)], and [(FAM–SFA) *vs*. (NAI–SC)]. The first two contrasts were performed to investigate the responses of FAM and NAI groups to both feeds. The comparison FAM-SC vs. NAI-SFA was performed to investigate the brain responses to two different unfamiliar feeds. In FAM-SC, the control C feed lacked the additive to which the animals were habituated. In NAI-SFA, the naive animals that were not habituated to the additive were exposed to it. This contrast was done to compare two situations: the disappearance of the familiar additive *vs*. the appearance of an unknown additive. The comparison FAM-SFA vs. NAI-SC was very important to the study. It consisted in comparing two situations where the animals were exposed to a familiar feed. The FA feed was as familiar to the FAM animals as was the C feed to the NAI animals. As a consequence, familiarity should not influence the brain responses observed, which would rather illustrate the added value of the FA *per se*.

We performed small volume correction (SVC) analyses on regions of interest (ROIs) in areas associated with encoding/expectation of reward, cognitive control, evaluation of food-related sensory stimuli and/or memory (anterior prefrontal cortex, dorsolateral prefrontal cortex, orbitofrontal cortex, insular cortex, cingulate cortex, entorhinal cortex, perirhinal cortex, prepyriform area, hippocampus, parahippocampal cortex, fusiform gyrus, putamen, caudate, globus pallidus, nucleus accumbens, amygdala). With this analysis, allowing for voxel to voxel comparisons within restricted ROIs, we managed to identify the voxels for which the activity was statistically different between groups/treatments in the ROIs. For the SVC analyses, the value of p = 0.05 (uncorrected for multiple comparisons) was set as the significance threshold with a cluster size *k ≥ 25* voxels.

The statistical analysis with SPM8 produced a listing of voxels for which the activation (CGM) differed between treatments. Each voxel was associated with a set of coordinates (x y z) corresponding to its spatial location in the *commissura anterior*-*commissura posterior* (CA-CP) plane with CP set as the origin. The ROIs chosen for the SVC analysis were anatomically identified on the basis of a 3D digitized pig brain atlas developed in our laboratory [16].

## Results

### Brain responses (glucose metabolism) to plain feed flavor and feed flavor with additive

The regions of differential glucose metabolism obtained with the SVC analyses are summarized in **Table 2**. In all contrasts, activated clusters were found in the (anterior and/or dorsolateral) prefrontal cortex and the left parahippocampal cortex. Different patterns of activations/deactivations were found in other brain structures.

**Table 2:** Summary of the brain activations (bold, for “a *vs*. b”, a > b) and deactivations (italic, for “a *vs*. b”, a < b) identified in the regions of interest selected for the Small Volume Correction (SVC) analysis using the SPM (Statistical Parametric Mapping) software (*t* > 1.71; p < 0.05 uncorrected, *k* ≥ 25 voxels) and for the following contrasts: familiarized group (FAM) *vs*. naive group (NAI), stimulation with feed flavor with additive (SFA) *vs*. control stimulation (SC), interaction between Familiarity and Feed stimulation (*F-*contrast), exposing NAI to SC *vs*. exposing NAI to SFA (effect of unknown additive in GC), exposing FAM to SFA *vs*. GFA vs. SC (effect of exposure to feed without additive in GFA), exposing FAM to SC *vs*. exposing NAI to SFA (comparing the exposure to the additive in naive animals and the exposure to the feed without additive in familiarized animals), exposing FAM to SFA *vs*. exposing NAI to SC (specific effect of SFA independent to habituation).

		Familiarity effect	Stimulation effect	Interaction effect	Exposure to unknown additive in NAI	Disappearance of known additive in FAM	The lesser of two evils	Feed additive effect
		FAM *vs*. NAI	SFA *vs*. SC	Familiarization × Feed Stimulation	NAI-SC *vs*. NAI-SFA	FAM-SFA *vs*. FAM-SC	FAM-SC *vs*. NAI-SFA	FAM-SFA *vs*. NAI-SC
Cortical structures									
aPFC	L	**4.45 (-0 42–3)**	**2.22 (-4 33–5)**		**4.24 (-6 34 4)**	**5.55 (-0 42 2)**	**3.17 (-2 42–4)**	**3.86 (-2 41–4)**
aPFC	R	**4.45 (0 42–3)**				**8.14 (0 42 2)**	**2.88 (0 42–3)**	**5.50 (2 43–3)**
dlPFC	L	**2.79 (-2 27 20)**			**3.29 (-8 41 9)**		**3.68 (-4 40 11)**	**2.69 (-2 28 20)**
dlPFC	R	**2.40 (2 27 20)**				**5.61 (10 44 5)**		
OFC	L							
OFC	R							
IC	L	**2.55 (-22 14 13)**	**2.62 (-16 35 5)**		*4*.*08 (-8 33 4)*	**2.91 (-12 37 2)**		**3.50 (-22 15 14)**
IC	R	**2.26 (22 10 15)**	**2.59 (20 20 7)**	11.82 (14 32 8)		*2*.*85 (14 31 8)*	*2*.*60 (20 20 9)*	*2*.*29 (14 33 8)*
							**2.71 (20 10 16)**	
daCC	L							
daCC	R							
dpCC	L		*3*.*55 (-4–12 11)*	6.71 (-0 1 18)	**5.27 (-6–12 12)**	*2*.*88 (-2–1 20)*	**2.30 (-8–12 10)**	
dpCC	R			2.21 (4 14 15)				
vaCC	L							
vaCC	R							
vpCC	L							
vpCC	R							
aEC	L		**2.64 (-18 0–1)**			**2.54 (-16 3–3)**		
aEC	R		**2.21 (16–3 0)**	7.48 (14–3 1)	*3*.*32 (14–3 1)*			
PC	L		*3*.*30 (-4–10 10)*			*3*.*22 (-4–9 13)*		
PC	R		**1.91 (12 5–7)**			**3.92 (10 7–6)**		
PPA	L		**3.14 (-18 11 1)**		*2*.*46 (-16 13 7)*	**3.31 (-10 34–5)**		**2.55 (-18 11 0)**
PPA	R		**3.66 (18 12 1)**			**2.87 (16 11–2)**		**4.79 (16 11–1)**
HIP	L							
HIP	R					**5.12 (12 7–5)**		
PHC	L	**3.01 (-4–17 18)**	**3.18 (-22–1 1)**		**6.33 (-8–17 11)**	**2.34 (-16–4 0)**	**2.54 (-4 17 17)**	**3.04 (-20–2 1)**
			*3*.*67 (-6–12 10)*				*2*.*00 (-22 8 5)*	*2*.*50 (-6–13 9)*
PHC	R		**2.95 (22 0 1)**		*2*.*97 (20 9 0)*	**2.26 (22 2 0)**	*2*.*91 (18–5–1)*	
FG	L							
FG	R							
Subcortical structures								
PUT	L	**3.21 (-16 6 6)**					**2.22 (-16 6 6)**	**3.96 (-16 7 7)**
								*2*.*43 (-6 20 1)*
PUT	R	**3.23 (14 10 4)**	**2.94 (14 11 4)**			*7*.*64 (8 22 6)*	*2*.*67 (10 16 2)*	**3.75 (14 10 4)**
		*3*.*54 (8 17 4)*	*2*.*17 (8 22 5)*					*4*.*53 (8 17 4)*
CAU	L	**2.71 (-4 17 8)**				*5*.*74 (-6 15 9)*	**3.59 (-4 18 8)**	*2*.*42 (-4 19 1)*
CAU	R	**3.06 (6 18 11)**					**3.21 (6 18 11)**	*3*.*73 (6 17 5)*
		*2*.*94 (6 17 5)*						
GP	L							
GP	R	*3*.*01 (8 16 2)*	**2.83 (12 12 2)**				*2*.*79 (8 16 2)*	
NA	L							
NA	R							
AMY	L		**2.91 (-18 7 4)**					**3.24 (-16 6 5)**
AMY	R	**3.57 (14 10 2)**	**3.84 (14 10 2)**		*3*.*21 (16 9 2)*	**4.46 (12 10–0)**		**5.42 (14 11–0)**

The peak *t*-value and coordinates in the CA-CP (*commissura anterior-commissura posterior*) reference plane are indicated for each significant cluster, for the left (L) and right (R) hemispheres. aPFC, anterior prefrontal cortex; dlPFC, dorsolateral prefrontal cortex; OFC, orbitofrontal cortex; IC, insular cortex; daCC, dorsal anterior cingulate cortex; dpCC, dorsal posterior cingulate cortex; vaCC, ventral anterior cingulate cortex; vpCC, ventral posterior cingulate cortex; aEC, anterior entorhinal cortex; PC, perirhinal cortex; PPA, prepyriform area; HIP, hippocampus; PHC, parahippocampal cortex; FG, fusiform gyrus; PUT, putamen; CAU, caudate nucleus; GP, globus pallidus; NA, nucleus accumbens; AMY, amygdala. Representative images from the last 4 contrasts are showed in **Figs 3** and **4**.

Compared to naive piglets, piglets familiarized with the FA from weaning had significant brain activations in the insular cortex, the amygdala and the striatum (putamen and caudate). Deactivated clusters were also found in the striatum (putamen, caudate and globus pallidus), but in the right hemisphere only.

Compared to the stimulation with the control feed flavor, the stimulation with the FA feed flavor triggered activations in the insular, entorhinal and perirhinal cortices, the prepyriform area, the amygdala and the right striatum (putamen and globus pallidus). Significant clusters of deactivations were also found in the perirhinal, parahippocampal and cingulate cortices, and the putamen, but in the left hemisphere only.

**Fig 2:**
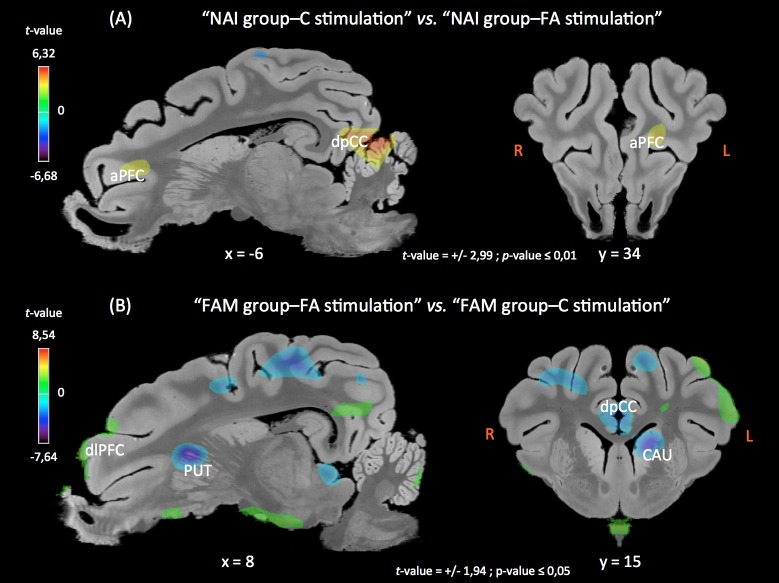
**Sagittal and coronal (R: right, L: left) MRI sections showing clusters of differential glucose metabolism for 2 contrasts: “NAI group-C stimulation” *vs*. “NAI group-FA stimulation” (top panel), and “FAM group-FA stimulation” *vs*. “FAM group-C stimulation” (bottom panel).** Clusters were identified during the SVC (Small Volume Correction) analyses in different regions of interest chosen upon *a priori* hypotheses. The x or y coordinates in the CA-CP (*commissura anterior-commissura posterior*) plane and the thresholds for significance (uncorrected) are indicated below the images. Positive *t*-values (orange-yellow-green) indicate a glucose metabolism higher in “NAI group-C stimulation” compared to “NAI group-FA stimulation”, and in “FAM group-FA stimulation” compared to “FAM group-C stimulation”. Negative *t*-values (purple-blue) indicate a glucose metabolism lower in “NAI group-C stimulation” compared to “NAI group-FA stimulation”, and in “FAM group-FA stimulation” compared to “FAM group-C stimulation”. aPFC, anterior prefrontal cortex; CAU, caudate nucleus; dlPFC, dorsolateral prefrontal cortex; dpCC, dorsal posterior cingulate cortex; PUT, putamen.

In the naive group, compared to the perception of the FA feed flavor, the perception of the control feed flavor triggered activations in the left cingular cortex, and deactivations in the right amygdala, entorhinal, and parahippocampal cortices, as well as in the left prepyriform area and insular cortex. Unlike other contrasts, no clusters of differential activations were found in the striatum in this contrast (**Fig 2A**).

In the familiarized group, compared to the perception of the control feed flavor, the stimulation with the FA feed flavor triggered activations in the left insular cortex, the entorhinal and perirhinal cortices, the prepyriform area, the hippocampus, and the amygdala, and deactivations in the right insular cortex, the cingular and perirhinal cortices, and the striatum (putamen and caudate; **Fig 2B**).

**Fig 3:**
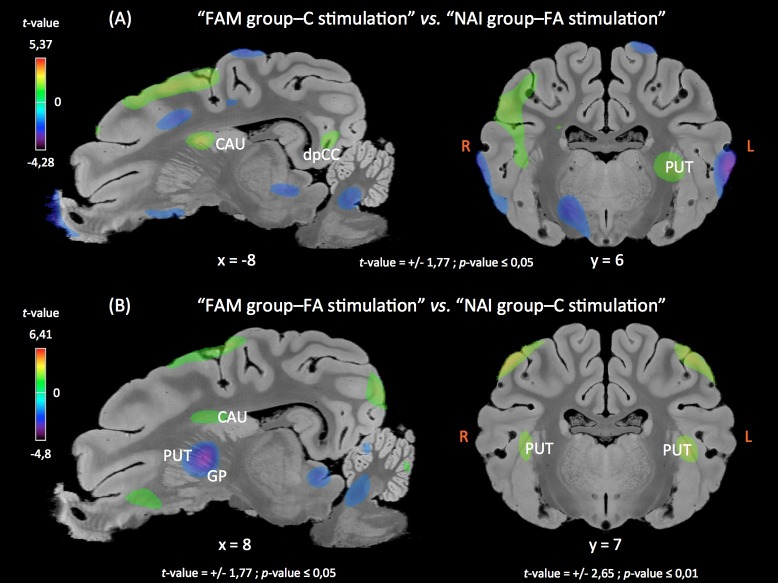
**Sagittal and coronal (R: right, L: left) MRI sections showing clusters of differential glucose metabolism for 2 contrasts: “FAM group-C stimulation” *vs*. “NAI group-FA stimulation” (top panel), and “FAM group-FA stimulation” *vs*. “NAI group-C stimulation” (bottom panel).** Clusters were identified during the SVC (Small Volume Correction) analyses in different regions of interest chosen upon *a priori* hypotheses. The x or y coordinates in the CA-CP (*commissura anterior-commissura posterior*) plane and the thresholds for significance (uncorrected) are indicated below the images. Positive *t*-values (orange-yellow-green) indicate a glucose metabolism higher in “FAM group-C stimulation” compared to “NAI group-FA stimulation”, and in “FAM group-FA stimulation” compared to “NAI group-C stimulation”. Negative *t*-values (purple-blue) indicate a glucose metabolism lower in “FAM group-C stimulation” compared to “NAI group-FA stimulation”, and in “FAM group-FA stimulation” compared to “NAI group-C stimulation”. CAU, caudate nucleus; dpCC, dorsal posterior cingulate cortex; PUT, putamen.

Clusters of higher activations were found in the right insular cortex, the cingular cortex and the striatum (putamen and caudate) in the familiarized pigs perceiving the control feed flavor than in the naive pigs perceiving the feed flavor added with the unfamiliar FA. Deactivated clusters were found in the right insular cortex, the parahippocampal cortex, and the right striatum (putamen and globus pallidus; **Fig 3A**).

**Fig 4:**
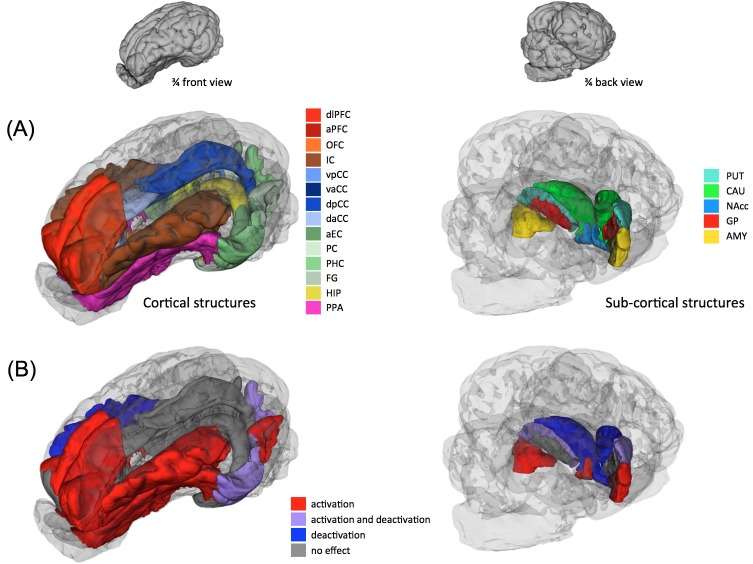
**Three-dimensional representations of the (A) cortical and subcortical brain structures of interest, and (B) their metabolism modifications in the contrast “FAM group-FA stimulation” compared to “NAI group-C stimulation”.** The cortical structures are shown in a ¾ front view of the brain 3D model (the total brain appears transparent). The subcortical structures are shown in a ¾ back view. dlPFC, dorsolateral prefrontal cortex; aPFC, anterior prefrontal cortex; OFC, orbitofrontal cortex; IC, insular cortex; vpCC, ventral posterior cingulate cortex; vaCC, ventral anterior cingulate cortex; dpCC, dorsal posterior cingulate cortex; daCC, dorsal anterior cingulate cortex; aEC, anterior enthorinal cortex; PC, perirhinal cortex; PHC, parahippocampal cortex; FG, fusiform gyrus; HIP, hippocampus; PPA, prepyriform area; PUT, putamen; CAU, caudate nucleus; NA, nucleus accumbens; GP, globus pallidus; AMY, amygdala. In panel (B), higher activation in “FAM group-FA stimulation” compared to “NAI group-C stimulation” appears in red, lower activation appears in blue, ambivalent responses (activation and deactivation in different sub-regions of a structure) appear in purple, and no difference between treatments appears in grey.

Compared to the perception of the familiar control feed flavor by the naive pigs, the perception of the familiar FA feed flavor by the familiarized pigs induced activations in the left insular cortex, the prepyriform area, the amygdala and the dorsal putamen, and deactivations in the right insular cortex, the parahippocampal cortex, and the ventral putamen and caudate (**Fig 3B**). A three-dimensional representation of the brain structures of interest and their metabolism modifications in this contrast are provided in Fig 4.

### Feed intake, weight gain and feed efficiency

No significant differences were observed between groups for the ADG, ADFI, and G:F (**Table 3**).

**Table 3:** Early post-weaning (1 to 29 days)^1^ performance of piglets exposed (familiarized) or not (naive) to a feed additive from weaning to 28 days after weaning.

		Treatment groups		
		FAM^2^	NAI^2^	p-value^3^
BW, kg				
	Initial BW (d 1)	8.86 ± 0.21	8.75 ± 0.21	0.707
	Final BW (d 29)	23.31 ± 0.84	21.66 ± 0.84	0.135
ADG, g/d				
	d 1–28	516	461	0.146
	d 1–7	248	273	0.494
	d 8–14	686	623	0.460
	d 15–21	504	439	0.522
	d 22–28	627	509	0.119
ADFI, g/d				
	d 1–28	684	659	0.608
	d 1–7	278	311	0.289
	d 8–14	735	712	0.752
	d 15–21^4^	843	813	0.720
	d 22–28^4^	1031	907	0.250
G:F, g/g				
	d 1–28	0.76	0.70	0.053^5^
	d 1–7	0.88	0.87	0.751
	d 8–14	0.93	0.87	0.148
	d 15–21	0.58	0.51	0.465
	d 22–28	0.64	0.56	0.376

^1^ Pigs per treatment: *n* = 8. The feed additive was added in a standard pre-transition diet from d 1 (weaning) to d 15 and in a standard post-transition diet from d 16 to d 28.

^2^ FAM, familiarized with the feed additive; NAI, naive, not exposed to the feed additive.

^3^ One-way ANOVA among treatments.

^4^ Total feed intake was not assessed on 2-choice test days (d 16 and d 23). Consequently, ADFI was averaged without these data.

^5^ Trends were considered at 0.05 < p ≤ 0.10.

### Feed preferences

**Fig 5:**
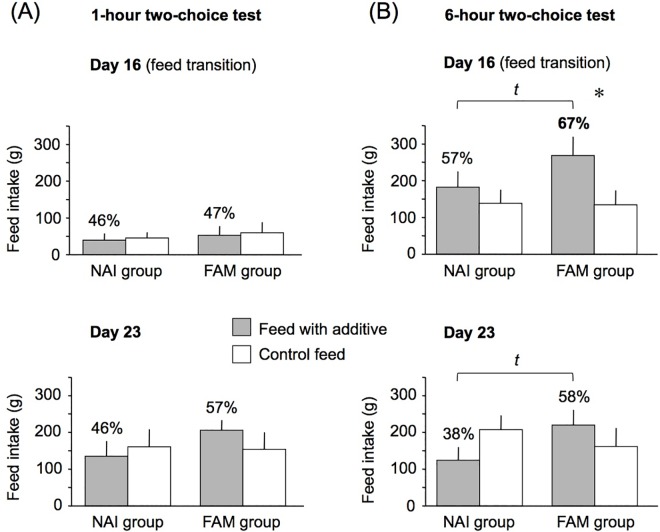
**Feed intake (g) during the (A) 1-h 2-choice feeding tests and the (B) 6-h 2-choice feeding tests in the naive (NAI) and familiarized (FAM) groups.** All animals were fed a first stage pre-transition diet from weaning to d 15, with (FA) or without (C) additive. On d 16, a feed transition was performed and the pre-transition diet was replaced by a post-transition diet of different composition, with (FA) or without (C) the same additive. During the 2-choice tests performed on d 16 and d 23, the piglets had the choice between the post-transition diet without additive and the same diet with the additive. Data are represented with means ± SEMs. Percentages in bars indicate diet intake relative to total intake. *t* indicates trend for the FAM group to consume more FA feed than the NAI group during the 6-h tests on d 16 (p = 0.14) and d 23 (p = 0.10). The asterisk indicates a moderate preference for the feed, *i*.*e*. a percentage of feed intake from 60 to 80% of total feed intake, according to the scale of Goatcher and Church described in Kennedy and Baldwin [15].

Overall, in both groups the consumption of the FA feed and C feed during the 1-h and 6-h 2-choice feeding tests performed on d 16 and d 23 did not significantly differ (p ≥ 0.10 for all; **Fig 5**). In the familiarized group, the intake of FA feed during the 6-h test performed on d 16, *i*.*e*. the day of feed transition, represented 67% of total feed intake, suggesting a moderate preference for the FA feed in this group [15]. In the naive group, the intake of the familiar control feed during the 6-h test on d 23 represented 62% of total intake (FA: 38% of total intake), suggesting a moderate preference for the control feed in this group.

## Discussion

The aim of our study was to demonstrate that the sensory perception of a feed additive (FA) can modulate brain activity in areas involved in hedonic and motivational processes or in learning and memory, in relation to and/or independently from familiarization with this additive in the pig model. Overall, our results indicated that piglets familiarized with FA from weaning (familiarized piglets) had increased brain responses compared to naive piglets in the prefrontal, insular, and parahippocampal cortices. Their brain metabolism also differed in the striatum and amygdala. Piglets exposed to FA feed flavor during brain imaging exhibited different brain responses than piglets exposed to the control feed flavor (in the aforementioned brain areas plus the cingulate, entorhinal and perirhinal cortices, the amygdala and the prepyriform area), and notably an increased activation of the insular cortex and the amygdala. There was also a significant effect of interaction between familiarity with the additive and the type of feed stimulation on brain responses in the insular and cingulate cortices.

Even more interesting are the results from the pairwise comparisons demonstrating that 1) the perception of the FA feed flavor by the naive piglets (**Fig 2A**) modulated brain glucose metabolism in cortical areas and the amygdala but not in the striatum, suggesting that the additive is not likely to stimulate the brain reward circuit without habituation; 2) the perception of the control feed flavor by the familiarized piglets (**Fig 2B**) modulated activity in cortical and subcortical (including striatal) structures, meaning that familiarized piglets can discriminate between feed flavors with and without the additive and that, in familiarized piglets, the brain reward circuit is impacted by the presence of the additive in the feed flavor; 3) brain responses to unfamiliar feeds were not the same in naive and familiarized piglets (**Fig 3A**), *i*.*e*. the naive piglets did not respond to the unfamiliar FA feed like the familiarized piglets responded to the unfamiliar control feed, suggesting that adding or removing a flavor from a familiar feed basis does not impact the hedonic circuit similarly; and 4) brain responses to familiar feeds were not the same in naive and familiarized piglets (**Figs 3B** and **4**), *i*.*e*. naive piglets did not respond to the control feed like familiarized piglets responded to the FA feed.

The sensory feed additive used in this study was previously shown to increase feed intake and motivation, especially after a feed transition [10]. In the present study, we did not report any significant improvement of production traits, but found that the piglets that were familiarized with the FA showed a moderate preference for the post-transition feed with the additive compared to the control feed without additive on the day of transition (67% of total feed intake). Indeed, according to the scale of Goatcher and Church described in Kennedy and Baldwin [15], a percentage of feed intake from 60 to 80% of total feed intake indicates a moderate preference for the feed. As previously suggested by our group [10], palatability perception is thought to be subjected to a large inter-individual variability, which might explain the lack of clear-cut/systematic preferences. We assume that using more animals would increase the statistical power and enable to confirm or disprove the preference for the FA feed in the familiarized group.

The moderate preference for the FA feed observed only in the pigs that were familiarized to the additive from weaning onwards seems to be supported by the brain imaging data. Indeed, the only contrast for which no difference was found in the striatum was the comparison between control and FA feed stimulation in the naive group, which indicates that perception of the FA feed flavor without previous exposure to the additive triggered no spontaneous activation of the brain reward circuit, suggesting that chronic pre-exposure is necessary to activate this neural network when the FA flavor is perceived. Interestingly, the fact that the FA flavor was relatively new in naive animals (the FA diet was offered only twice at d 16 and d 23 during the two-choice feeding tests) was not sufficient to deactivate the brain reward circuit, as previously observed with aversive flavors [8]. The results from the 2-choice test on the day of feed transition support the brain imaging data and confirm that the naive animals exhibited no neophobic responses for the unknown FA, since the intake of the FA feed during the first hour of the test was 46% of total feed intake, and 57% of total feed intake after 6 hours.

Interestingly, the anterior and/or dorsolateral prefrontal cortices, as well as the left parahippocampal cortex, were systematically activated in the group exposed to its familiar feed flavor (control feed for naive animals, FA feed for familiarized animals). Prefrontal cortex (PFC) areas are linked to cognitive evaluation processes, such as evaluation of rewarding stimuli, and explicit memory regions [17, 18]. The activation of the PFC in our animals was therefore dependent on the familiarity with the feed flavor. According to this postulate, it is not surprising to also observe greater PFC activity in familiarized animals exposed to the control feed flavor than in naive animals exposed to the FA feed flavor, because the feed flavor, even without the FA flavor, is more familiar to the familiarized animals than the unfamiliar FA flavor to naive animals. The addition of the FA flavor could also cover the familiar feed flavor in control animals.

The amygdala, left insular cortex, and prepyriform area were only activated in familiarized animals exposed to the FA feed flavor. The amygdala, insular cortex, and hippocampal formation are important for food recognition and taste processing [18–20], while the prepyriform area, the traditional primary olfactory cortex, is the largest cortical recipient of afferent fibers from the olfactory bulb and is assumed to play an important role in olfactory learning tasks [21, 22]. It is very interesting to note that the taste pleasantness of a food stimulus predicted left insula response in humans [23], and that the insular cortex activity was modified by the perception of pleasant food stimuli (sweet) in pigs [18]. Activation in these structures exclusively during stimulation with FA in familiarized animals may indicate that the familiar feed with FA in familiarized animals is not perceived as the familiar control feed in naive animals, and that some responses in brain regions involved in sensory and hedonic processes are specific to the FA and independent to familiarity factors.

These data suggest that the FA feed flavor activated different memory circuits than the feed flavor without FA, a hypothesis that seems supported by the results from the contrast comparing the respective familiar feed flavors in naive and familiarized animals. Indeed, the control feed is as familiar to the naive animals than the FA feed is familiar to the familiarized animals. Yet, both these familiar feeds did not activate the dorsal striatum similarly. The dorsal putamen was bilaterally activated in familiarized animals exposed to the FA feed compared to naive animals exposed to the control feed, but the ventral putamen and caudate were deactivated. In brain imaging studies using food stimuli in humans and animals, activation and deactivation of the dorsal striatum (*via* dopamine (DA) signaling or BOLD signal) are consistently interpreted as part of the anticipation and perception of pleasure, respectively [24–26]. Small *et al*. [27] also demonstrated in the human that feeding is associated with DA release in the dorsal putamen and caudate, but not in the ventral striatum, and that the amount of DA released correlates with meal pleasantness. It is important to note that no activation of the ventral striatum (nucleus accumbens) was found in our animals. The bilateral activation of the dorsal putamen might indicate more pleasure anticipated by the perception of the familiarized FA feed flavor in the familiarized animals than by the perception of the control feed flavor in the naive animals (**Figs 3** and **4**).

The coexistent deactivation of the putamen and caudate is trickier to explain. It might either be interpreted as 1) the perception of pleasure triggered by food stimuli receipt, or 2) a response to a negative or ambivalent aspect of the stimulation. Indeed, the diffusion of odor and taste is more than a mere predictive cue (*e*.*g*. visual stimulus) but less than food receipt that includes post-ingestive signals. It is consequently possible that both reward anticipation and perception processes occurred in our animals, illustrating both activations and deactivations in different parts of the dorsal striatum. We previously demonstrated in pigs that appetitive oral stimuli only (without visceral stimulation) could induce both activations and deactivations in different parts of the dorsal striatum [18]. Moreover we consistently found in previous studies that the perception of a preferred flavor compared to an aversive or less preferred flavor triggered different activations or deactivations in the left and right hemispheres [7, 8]. Interestingly, Henkin & Levy [28] suggested that the left hemisphere is involved in emotional processing of odors and hedonic judgments, while the right hemisphere is rather involved in the processing of odor familiarity and recognition. The second hypothesis is that, in addition to the positive perception of the FA (incentive salience), something might have been perceived as negative. In a previous study, we reported that higher concentrations of the FA in the food than that used in this study is aversive [9], which suggests that the concentration of FA is susceptible to alter its intrinsic hedonic value. As mentioned in the *Materials and Methods*, the FA matrix used in the feed beverages was different than that used in the solid feed, and the concentration was calculated to be similar in both vectors. Nevertheless, it is possible that the FA volatile compounds have a higher diffusion coefficient in a liquid matrix than in a solid matrix, consequently increasing the perceived concentration of FA in the feed solution. Further developments are necessary to solve this question and precisely control the perceived concentration of FA in different matrices (*e*.*g*. solid food, liquid, or air).

The FA used in this study is composed of a natural extract of *Citrus sinensis* (60–80%). Sweet orange belongs to the Hesperide family (citrus), which also includes the bitter orange, lemon, lime, grapefruit, and bergamot for example. Amongst the Hesperide main active compounds are monoterpenes, monoterpenic aldehydes, and flavonoids. Several studies investigated the behavioral and neurophysiological effects of citrus aromas and extracts. One major effect described in the literature is the decrease of anxiety symptoms. In the human, sweet orange aromas can decrease the anxiety state and subjective psychological tension during an anxiogenic experimental situation [29]. Orange odor can also reduce the anxiety level, heart rate and salivary cortisol in patients at the dentist [30, 31]. Air diffusion of essential oils of bergamot can decrease arterial pressure and heart rate, as well as increase the vagosympathetic balance in high-stress workers [32]. Citrus fragrance even normalized neuroendocrine hormone levels and immune function in depressive subjects, which permitted to markedly reduce the doses of antidepressants in these patients [33]. Comparable results were obtained in rodents. Studies in mice demonstrated that citrus fragrance reduced immobility time in the forced swimming test [34] and accelerated the metabolic turnover of DA in the hippocampus and of serotonin (5-HT) in the prefrontal cortex and striatum [35]. Mice confronted to anxiogenic situations increased their exploratory behavior when exposed to sweet orange aromas [36]. Similarly, rats subjected to an anxiogenic situation increased their locomotor and exploratory activity when exposed to essentials oils of bergamot, while their corticosterone levels decreased and their glutamate release in the hippocampus increased [37, 38].

Citrus components can also modulate eating behavior and appetite. Interestingly, Yi *et al*. [39] tested the effects of apigenin in rats subjected to chronic mild stress (CMS), and showed that this bioflavonoid of the citrus group reduced immobility time and the usual decrease in food ingestion, while attenuating the CMS-induced alterations in 5-HT and DA levels in distinct brain regions. The scent of essential oil of grapefruit affected the autonomic neurotransmission and blood pressure through a histaminergic response, and reduced appetite and body weight in rats [40, 41]. Other studies suggested that bitter orange (*Citrus aurantium*) might have appetite-suppressing properties for body weight control (for review, see [42, 43]), but sweet orange extracts supplementation increased learned and spontaneous feed preferences in lambs and piglets [10, 44].

Too few studies investigated the effects of citrus extracts on eating behavior, but on the basis of the aforementioned literature mainly focused on anxiety and stress, we can assume that if some components of the citrus group can modulate appetite, these effects are more likely to appear in anxiogenic situations that usually alter eating behavior and motivation (*e*.*g*. depressing environment or stressful conditions, behavioral tests, food transitions). As a consequence, further studies are needed to explore the potential of citrus extracts to stimulate appetite during stressful events (*e*.*g*. socio-environmental or food transitions) or in fragile populations (*e*.*g*. in the elderly or in patients with appetite disorders). But because different citrus extracts induced different effects in the literature (*e*.*g*. decreased appetite with bitter orange compared to increased food preference with sweet orange), different components (*e*.*g*. apigenin, limonene, etc.) present at different rates in different citrus fruits [45, 46] might have different effects on eating behavior and reward. To complicate the matter, similar extracts might also have different effects depending on their ambient concentration or inclusion rate in the food. As previously stated, the sweet-orange-based food additive used in the present study, which increased food preference in Clouard and Val-Laillet [10], rather induced food aversion at higher doses [9]. Finally, we cannot exclude the possibility that other active compounds than citrus contained in the FA are responsible for the effects observed on hedonic brain responses in our study. In further studies, it would be interesting to investigate the brain responses to a FA with a different ingredient composition. For example, we could use another FA amongst those that were tested in a previous behavioral study and for which significant effects were found [10].

Finally, it worth noting that, in our study, we investigated brain responses to feed flavor in 8- to 9-week-old pigs, *i*.*e*. before the end of brain development occurring around 24 weeks of age in pigs [47, 48]. Although we previously reported brain responses similar to those found in human adults in juvenile pigs of about 10–12 weeks of age, *i*.*e*. also during brain development [7, 8, 18], we cannot rule out the possibility that brain responses would be different in animals with mature brain, suggesting that our findings should be replicated in older pigs before further extrapolation of research results to adult individuals.

In conclusion, our results demonstrated that the sweet-orange-based FA used in this study induced specific brain responses, including responses potentially related to reward anticipation and perception processes, in the individuals familiarized to it. This effect, however, was not obtained with a similarly familiar feed flavor without FA in naive animals, suggesting that chronic exposure to the FA is necessary to obtain positive effects in terms of hedonism, but familiarity only cannot explain the effects of the FA on brain responses. The postulate of a “hedonic added value” of the FA suggested by the brain imaging data requires further investigation as it was not supported by the behavioral feed preference data in the present study. In regard to the literature on citrus extracts, fragile individuals or populations subjected to stress or anxiety, as well as appetite disorders, should be preferentially targeted. Further work is needed to identify the optimal concentrations according to the vector matrices, the active compounds responsible for the positive effects on mood and appetite, and the neurobiological mechanisms (including neurotransmitters systems) involved in these effects on brain metabolism and behavior.
